# Evaluation of Oxidative Stress Biomarkers, Pro-Inflammatory Cytokines, and Histological Changes in Experimental Hypertension, Dyslipidemia, and Type 1 Diabetes Mellitus

**DOI:** 10.3390/ijms23031438

**Published:** 2022-01-27

**Authors:** Paul-Mihai Boarescu, Ioana Boarescu, Raluca Maria Pop, Ştefan Horia Roşian, Ioana Corina Bocșan, Vasile Rus, Răzvan Olimpiu Mada, Iulia Diana Popa, Nicholas Neagu, Adriana Elena Bulboacă, Anca Dana Buzoianu, Sorana D. Bolboacă

**Affiliations:** 1 Department of Pharmacology, Toxicology and Clinical Pharmacology, Iuliu Haţieganu University of Medicine and Pharmacy Cluj-Napoca, Gheorghe Marinescu Street, No. 23, 400337 Cluj-Napoca, Romania; boarescu.paul@umfcluj.ro (P.-M.B.); raluca.pop@umfcluj.ro (R.M.P.); bocsan.corina@umfcluj.ro (I.C.B.); abuzoianu@umfcluj.ro (A.D.B.); 2 Department of Medical Informatics and Biostatistics, Iuliu Haţieganu University of Medicine and Pharmacy Cluj-Napoca, Louis Pasteur Street, No. 6, 400349 Cluj-Napoca, Romania; ioana.chirila.boarescu@elearn.umfcluj.ro (I.B.); sbolboaca@umfcluj.ro (S.D.B.); 3 Department of Cardiology—Heart Institute, “Iuliu Haţieganu” University of Medicine and Pharmacy Cluj-Napoca, Calea Moților Street, No. 19–21, 400001 Cluj-Napoca, Romania; 4 “Niculae Stăncioiu” Heart Institute Cluj-Napoca, Calea Moților Street, No. 19–21, 400001 Cluj-Napoca, Romania; mada_razvan@yahoo.com (R.O.M.); diana.damian86@gmail.com (I.D.P.); 5 Department of Cell Biology, Histology and Embryology, University of Agricultural Sciences and Veterinary Medicine, Calea Mănăştur Street, No. 3–5, 400372 Cluj-Napoca, Romania; vasile.rus@usamvcluj.ro; 6 Faculty of Medicine, Iuliu Haţieganu University of Medicine and Pharmacy Cluj-Napoca, Luis Pasteur Street, No. 4, 400349 Cluj-Napoca, Romania; nicholasneagu1112@gmail.com; 7 Department of Pathophysiology, Iuliu Haţieganu University of Medicine and Pharmacy Cluj-Napoca, Victor Babeş Street, No. 2–4, 400012 Cluj-Napoca, Romania; adriana.bulboaca@umfcluj.ro

**Keywords:** atherosclerosis, inflammation, oxidative stress, hypertension, dyslipidemia, type 1 diabetes mellitus

## Abstract

The present study aims to compare the oxidative stress biomarkers, pro-inflammatory cytokines, and histological changes induced by three cardiovascular risk factors, namely, hypertension, dyslipidemia, and type 1 diabetes mellitus. Hypertension was induced with 40 mg/kg body weight (b.w.) of N omega-nitro-L-arginine-methyl (L-NAME) administered orally. Dyslipidemia was induced by the administration of a diet with a high cholesterol (2%) content. Diabetes mellitus was induced by intraperitoneal administration of a single dose of streptozocin (65 mg/kg). Malondialdehyde (MDA) and total oxidative status (TOS) are increased by all three cardiovascular risk factors (up to 207%). The indirect assessment of NO synthesis (NOx) is observed to be reduced after L-NAME administration (43%), and dyslipidemia induction (16%), while type 1 diabetes mellitus is associated with the highest levels of NOx (increased 112%). Hypertension, dyslipidemia, and type 1 diabetes reduced the total antioxidative capacity (TAC) and total thiol (SH) levels (up to 57%). The values of evaluated pro-inflammatory cytokines, tumour necrosis factor-α (TNF-α), interleukin-6 (IL-6), and interleukin-1β (IL-1β), assessed from the ascending aorta were elevated by all three cardiovascular risk factors, with the highest levels induced by type 1 diabetes mellitus (up to 259%). The histopathological examination of the ascending and descending aorta revealed reversible pro-atherogenic changes consisting of the accumulation of lipid droplets in the subendothelial connective tissue on rats with hypertension and dyslipidemia. Irreversible pro-atherogenic changes consisting of a reduction of the specific elasticity of the arteries were observed in rats with type 1 diabetes mellitus. Type 1 diabetes mellitus demonstrates an alteration of the oxidative stress parameters, the elevation of tissue levels of the pro-inflammatory cytokines and causing irreversible pro-atherogenic changes on the aortic wall.

## 1. Introduction

Atherosclerosis is a major cause of vascular death. It is a progressive disease characterized by the accumulation of lipids and fibrous elements in the large arteries, developing into atheroma and characteristic plaques [1]. The acute rupture of these atheromatous plaques will cause partial or total occlusion of the affected artery due to local thrombosis. The clinical consequences of the rupture of the plaques will depend on their site and the degree and speed of vessel occlusion. The major clinical manifestations of atherosclerosis include ischemic stroke, ischemic heart disease, and peripheral arterial disease [1]. Ischemic heart disease often occurs at a lower prevalence rate than ischemic stroke and accounts for 10% to 35% of deaths [2].

Hypertension is a well-known risk factor for atherosclerosis. A positive relation between blood pressure values and atherosclerosis progression has been demonstrated for both systolic and diastolic blood pressure. It is supposed that elevated blood pressure leads to increased shear stress and turbulent blood flow, which eventually induces endothelial dysfunction and intimal injury. This can be a possible explanation for the increased atheroma formation at the arterial branches and areas with a higher turbulent flow [3].

Low-density lipoprotein cholesterol (LDL-chol) is the most abundant atherogenic lipoprotein in plasma, and it is also the primary source of cholesterol accumulated within the arterial wall in the atherosclerosis process. A strong relation was already demonstrated between the absolute changes in plasma LDL-chol levels and the risk of atherosclerotic process [4].

Atherosclerosis and cardiovascular disease prevalence are very high in patients with type 1 and type 2 diabetes mellitus (DM). Although patients with DM often associate with other cardiovascular risk factors such as hypertension and dyslipidemia, their cardiovascular risk is greater than what can be explained by these risk factors alone. Increased serum glucose levels favor glycosylation of plasma proteins, leading to endothelial dysfunction [3]. Endothelial dysfunction is an early event of atherosclerosis characterized by adhesion molecules, chemokines, leucocytes, increased low-density lipoprotein oxidation, platelet activation, and vascular smooth muscle cell proliferation and migration. The abnormal vascular phenotype represents an important risk factor that contributes to the pathogenesis of microvascular and macrovascular complications of diabetes [5,6]. Even more, hyperglycemia can also increase the permeability of the endothelial wall, facilitating the passage of pro-atherogenic molecules [3]. The early development of atherosclerosis was observed in young patients with type 1 DM [7]. The increased cardiovascular risk in the diabetic population is also associated with the development of diabetic cardiomyopathy. This cardiomyopathy is promoted by the long-standing metabolic perturbations of diabetes, thus exerting a direct toxic effect on the myocardium and occurring independently of other cardiac risk factors [8].

Oxidative stress represents one of the basic pathogenetic processes of atherosclerosis, as the increased production of reactive oxygen species (ROS) is closely related to endothelial dysfunction and the promotion of the vascular inflammatory response [9]. Common conditions that are also regarded as cardiovascular risk factors that predispose to atherosclerosis, such as hypercholesterolemia, hypertension, diabetes, and smoking, are associated with increased production of ROS [10].

Atherosclerosis is also recognized as an inflammatory disorder of the medium and large arteries. Cytokines have a profound influence on the pathogenesis of this disease as they are involved in all stages of atherosclerosis [11]. Tumour necrosis factor-α (TNF-α), interleukin (IL)-1, and IL-6 are pro-atherogenic cytokines secreted by macrophages, lymphocytes, natural killer cells, and vascular smooth muscle cells. TNF-α and IL-1 promote the expression of cytokines, adhesion molecules, and the migration and mitogenesis of vascular smooth muscle and endothelial cells on the vascular wall during the atherosclerotic process [12].

Rats have been used for a long time as animal models in studying cardiovascular diseases because specific characteristics of rats’ lipid metabolism are in-between those of humans and mice. Rats have a lower cost than larger animals like pigs and rabbits regarding purchasing, feeding, and maintenance. Compared to mouse models, rats have easier manipulation, blood collection, and dissection of blood vessels [13].

Most experimental studies of atherosclerosis in rats focus only on the following one cardiovascular risk factor: hypercholesterolemia. An old and straightforward model of atherosclerosis in rats consisted of the administration of a high cholesterol diet for an extended period of time (12 weeks) [14]. In contrast, new models are based on genetic modification and involve apolipoprotein E-deficient rats or low-density lipoprotein receptor-deficient rats [13].

The present study aimed to compare oxidative stress biomarkers, pro-inflammatory cytokines, and histological changes caused by the following three cardiovascular risk factors induced experimentally: hypertension, dyslipidemia, and type 1 diabetes mellitus.

## 2. Results

The analysis was conducted on all seven rats in the first three experimental groups (C, H, and D) and six in the DM experimental group.

### 2.1. Weight and Blood Pressure

**Figure 1:**
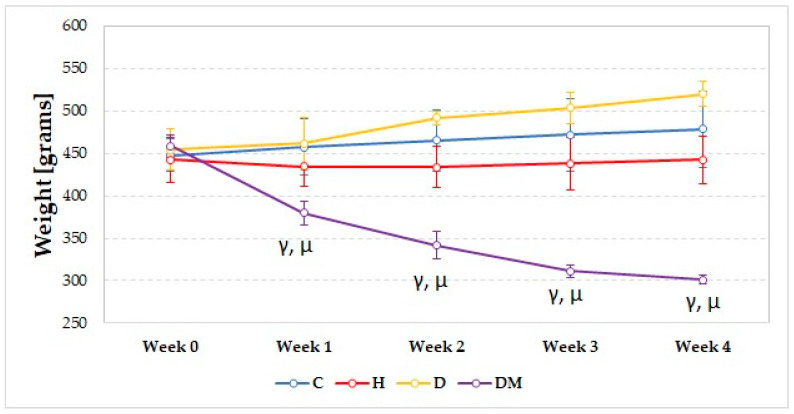
Evolution of weight of the rats; circles represent the median of each group. C = Control group, H = Hypertension group, D = Dyslipidemia group, DM = Type 1 Diabetes Mellitus group. The letter codes correspond to the *p*-values: < 0.020, ^γ^ DM compared to C, < 0.004, ^µ^ DM compared to D.

Diabetic rats associated weight loss compared to C and D groups, starting from the first week (*p* < 0.019, Figure 1). The weight significantly increased starting from the second week in the D group (Friedman test: *p* = 0.002) and significantly decreased in the DM group (Friedman test: *p* < 0.0001) (Figure 1).

**Figure 2:**
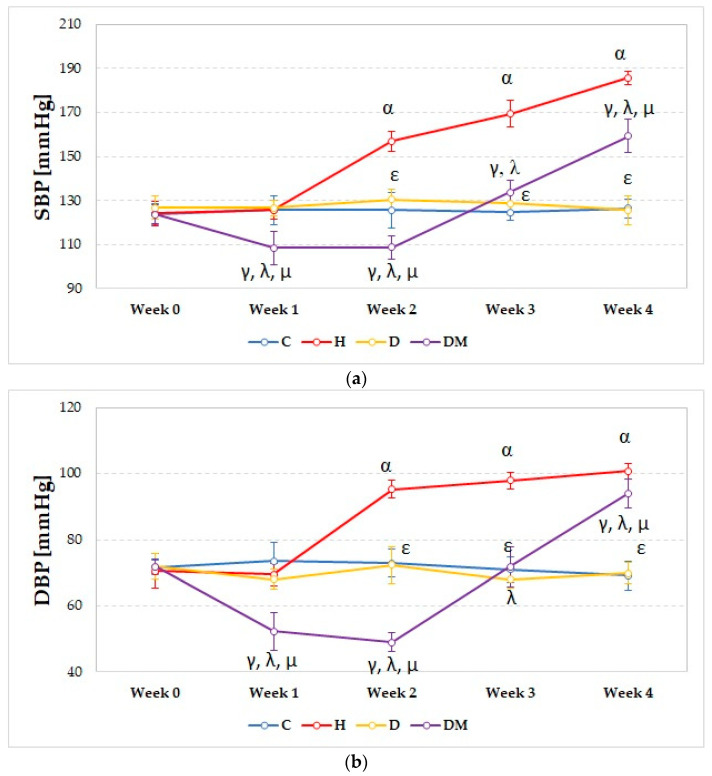
Evolution of: (**a**) Systolic blood pressure (SBP); (**b**) Diastolic blood pressure (SBP); Circles represent the median of each group. C = Control group, H = Hypertension group, D = Dyslipidemia group, DM = Type 1 Diabetes Mellitus group. The letter codes correspond to the *p*-values: < 0.035, ^α^ H compared to C, < 0.018, ^γ^ DM compared to C, < 0.016, ^ε^ D compared to H, < 0.035, ^λ^ DM compared to H, < 0.009, ^µ^ DM compared to D.

The rats from the H group presented elevated SBP and DBP compared to the C and DM groups, even from the second week (*p* ≤ 0.01, Figure 2a,b). Starting from the second week (*p* = 0.02, Figure 2a,b), significantly higher SBP and DBP were observed for the H group compared to the D group.

In diabetic rats, SBP and DBP were reduced compared to C and D groups in the first and second week (*p* ≤ 0.005, Figure 2a,b). In contrast, in the 4th week, both blood pressure values were elevated compared to these groups (*p* ≤ 0.003, Figure 2a,b).

Dyslipidemia did not significantly influence either SBP or DBP compared to the C group (Figure 2a,b).

### 2.2. Glucose and Lipid Profile

**Figure 3:**
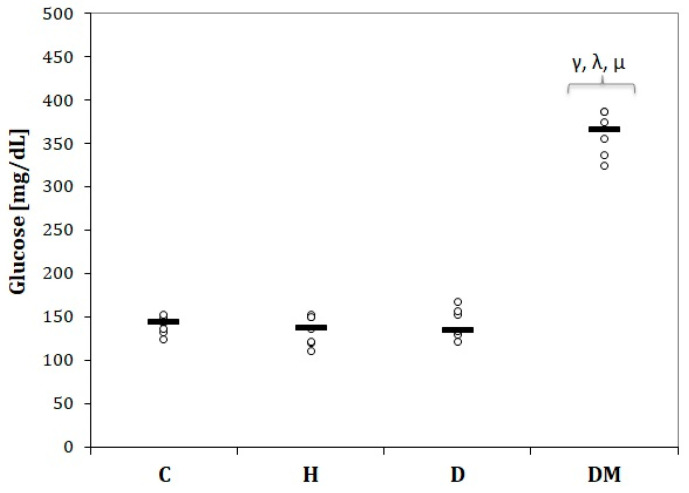
Serum glucose variation by groups; C = Control group, H = Hypertension group, D = Dyslipidemia group, DM = Type 1 Diabetes Mellitus group. The letter codes correspond to the Kruskal–Wallis post-hoc *p*-values: < 0.02, ^γ^ DM compared to C, < 0.004, ^λ^ DM compared to H, < 0.04, ^µ^ DM compared to D.

As expected, rats from the DM group were associated with the highest glucose levels (Figure 3). Neither the H group nor the D group influenced glucose levels compared to the C group (*p* > 0.05, Figure 3).

**Figure 4:**
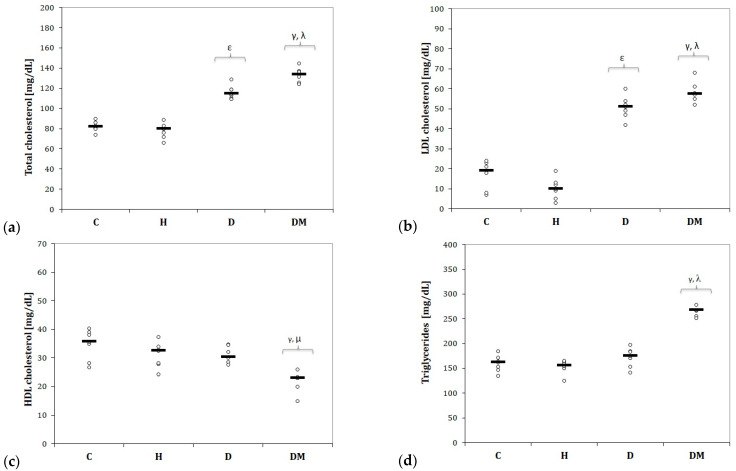
Variation of lipidic serum values by groups of: (**a**) Total cholesterol; (**b**) Low-density lipoprotein (LDL) cholesterol; (**c**) High-density lipoprotein (HDL) cholesterol; (**d**) Triglycerides. C = Control group, H = Hypertension group, D = Dyslipidemia group, DM = Type 1 Diabetes Mellitus group. The letter codes correspond to the Kruskal–Wallis post-hoc *p*-values: < 0.02, ^γ^ DM compared to C, < 0.025, ^ε^ D compared to H, < 0.003, ^λ^ DM compared to H, 0.046, ^µ^ DM compared to D.

Rats from the DM group presented increased total cholesterol levels due to increased LDL-chol levels (Figure 4a,b).

The serum levels of HDL-cholesterol were reduced, while triglycerides were increased in DM compared to all other groups (Figure 4c,d).

Rats from the D group presented increased levels of total cholesterol and LDL-chol levels (Figure 4a,b).

Dyslipidemia did not influence the HDL-cholesterol or triglyceride levels compared to the C group (*p* > 0.05, Figure 4c,d). Similar results were observed for rats with hypertension (*p* > 0.05, Figure 4c,d).

### 2.3. Oxidative Stress Parameters

**Figure 5:**
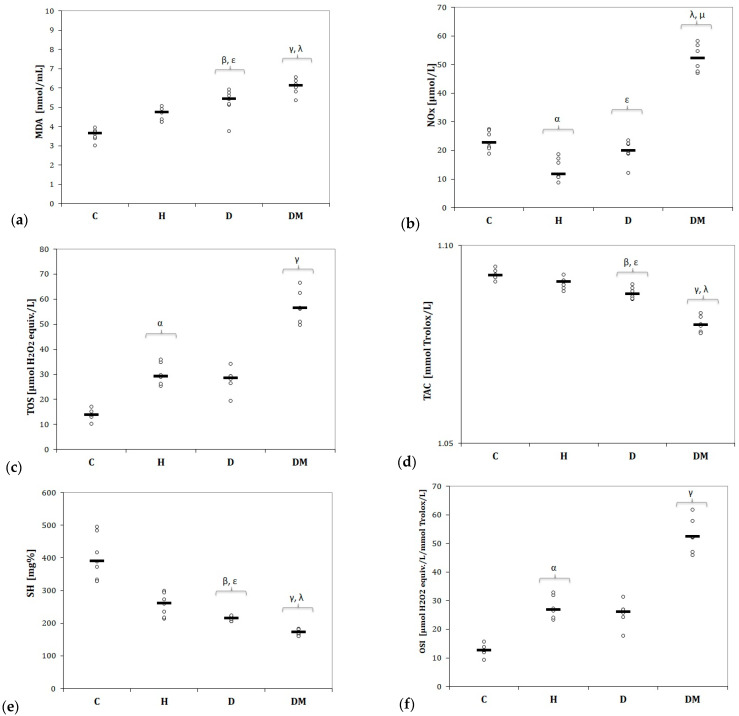
Variation of serum oxidative stress by groups of: (**a**) Malondialdehyde (MDA); (**b**) Nitric oxide (NOx); (**c**) Total oxidative status (TOS); (**d**) Total antioxidant capacity (TAC); (**e**) Thiols (SH); (**f**) Oxidative stress index (OSI). C = Control group, H = Hypertension group, D = Dyslipidemia group, DM = Type 1 Diabetes Mellitus group. The letter codes correspond to the Kruskal-Wallis post-hoc *p*-values: ^α^ H compared to C (0.035 NOx, 0.047 TOS, 0.047 OSI), ^β^ D compared to C (0.012 MDA, 0.020 TAC, 0.012 SH), ^γ^ DM compared to C (<0.001 MDA, TOS, TAC, SH, OSI), ^λ^ DM compared to H (0.049 MDA, <0.001 NOx, 0.011 TAC, 0.025 SH), ^µ^ DM compared to D (0.037 NOx).

The values of MDA increased in H, D, and DM groups with the highest levels for rats from the DM group (Figure 5a). The values of NOx were slightly reduced in the D group (*p* > 0.05, Figure 5b), and significantly reduced in the H group (*p* = 0.035, Figure 5b), compared to the C group. In rats with DM associated highest levels of NOx compared to the H and D groups (*p* < 0.04, Figure 5b). All experimental groups had elevated levels of TOS, but only the H and DM groups reached the significance threshold compared to C group (*p* = 0.047 for H and *p* < 0.0001 for DM group, Figure 5c). Rats from the DM group presented the most elevated parameter levels of the pro-oxidant parameters (*p* ≤ 0.003, Figure 5).

Hypertension, dyslipidemia, and diabetes mellitus reduced the antioxidant activity by decreasing TAC and total thiols (Figure 5d,e). Diabetes mellitus was the cardiovascular risk factor that reduced mostly TAC and SH compared to the other groups (*p* ≤ 0.018, Figure 5d,e).

According to our results, OSI was increased in all three experimental groups, especially in the H and DM groups (*p* ≤ 0.047, Figure 5f).

### 2.4. Pro-Inflammatory Cytokines

All tissue levels of pro-inflammatory cytokines were elevated in the H, D, and DM groups.

**Figure 6:**
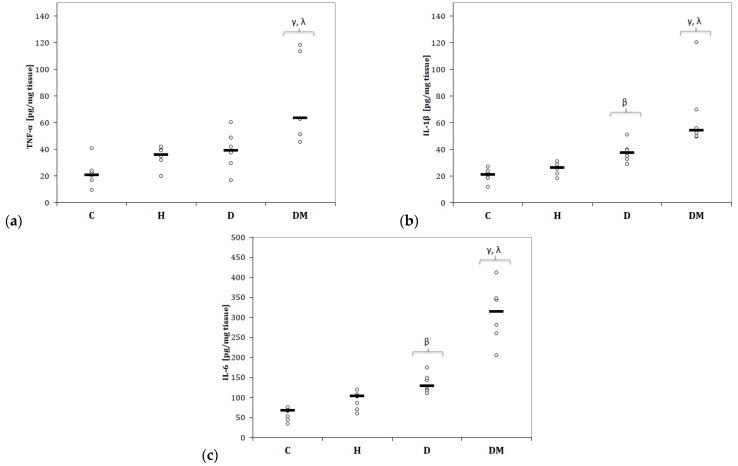
Variation of pro-inflammatory cytokines by groups of: (**a**) Tumour necrosis factor-α (TNF-α); (**b**) Interleukin 1β (IL-1β); (**c**) Interleukin 6 (IL-6). C = Control group, H = Hypertension group, D = Dyslipidemia group, DM = Type 1 Diabetes Mellitus group. Tumour necrosis factor-α (TNF-α), interleukin (IL)-1, and IL-6. The letter codes correspond to the Kruskal–Wallis post-hoc *p*-values: < 0.025, ^β^ D compared to C, < 0.001, ^γ^ DM compared to C, < 0.048 ^λ^ DM compared to H.

When compared to the C and H groups, rats in the DM group had the highest tissue levels of TNF-α (Kruskal–Wallis test: *p* = 0.0013), IL-1β (*p* = 0.0001), and IL-6 (*p* < 0.0001)) that were statistically significant in post-hoc analysis (Figure 6).

Rats with dyslipidemia (D group) presented higher tissue levels of evaluated cytokines compared to the C group (*p* ≤ 0.020, Figure 6), but when compared to the H group, IL-1β, and IL-6 were not significantly different (*p* > 0.25, Figure 6b,c).

In the H group, TNF-α, IL-1β, and IL-6 were slightly increased compared to the C group, but no statistical differences were found (*p* > 0.05, Figure 6a,b).

### 2.5. Histopathological Examination

**Figure 7:**
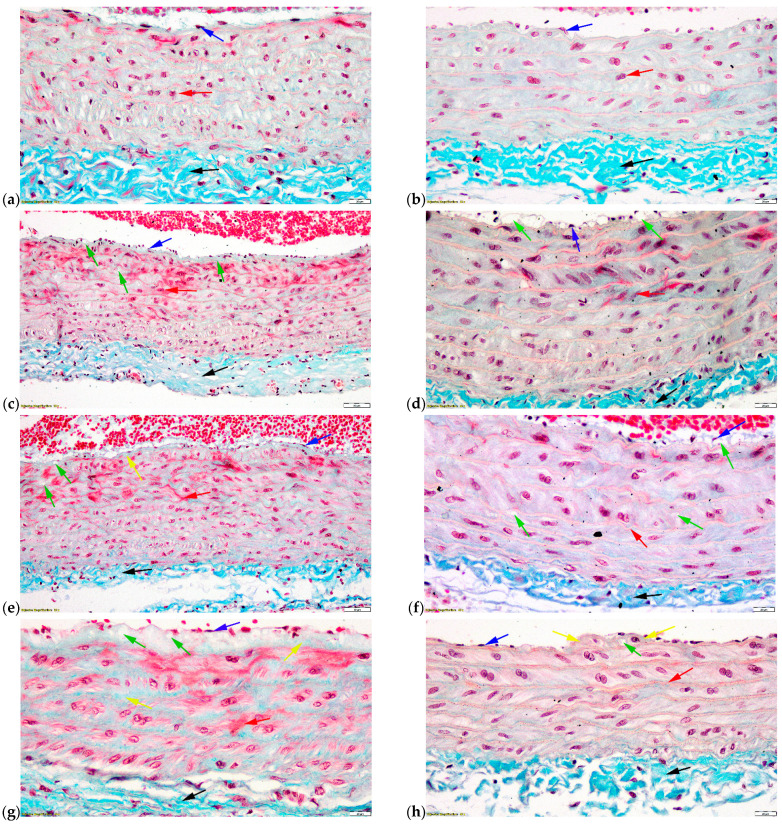
Histopathological examination of the aorta. (**a**) Control group—The base of the heart (the ascending aorta); (**b**) Control group—Descending aorta (the thoracic segment); (**c**) Hypertension group—The base of the heart (the ascending aorta); (**d**) Hypertension group—Descending aorta (the thoracic segment); (**e**) Dyslipidemia group—The base of the heart (the ascending aorta); (**f**) Dyslipidemia group—Descending aorta (the thoracic segment); (**g**) Type 1 diabetes mellitus group—The base of the heart (the ascending aorta); (**h**) Type 1 diabetes mellitus group—Descending aorta (the thoracic segment). Blue arrow—tunica intima; red arrow—tunica media; black arrow—tunica adventitia; green arrow—lipid droplets; yellow arrow—tendency to fibrosis.

Histopathological examination of the aorta, the base of the heart (the ascending aorta), and descending aorta (the thoracic segment) from all three groups is presented in Figure 7.

In the C group, the examined aortic fragments show normal aspects of the following three tunics specific to the blood vessels: tunica intima, tunica media, and tunica adventitia (Figure 7a,b). The ratio between the three tunics was normal, with the tunica intima representing a maximum of 5%, the tunica media about 80%, and the tunica adventitia about 15% of the artery wall.

In the H group, the only observed change was the accumulation of lipid droplets in the subendothelial connective tissue (Figure 7c,d). In the structure of the tunica intima of the ascending aorta, rare small-to-medium-sized lipid droplets were observed. The lipid droplets present in the tunica intima are better represented in the descending aorta than in the ascending aorta.

In the ascending aorta of the rats from the D group, the degree of lipid droplets accumulation in the subendothelial connective tissue seemed lower than in the H group. Moreover, a thickening of the subendothelial connective tissue with a tendency to fibrosis was also observed (Figure 7e). In the tunica media of the ascending aorta, the lipid droplets are slightly more numerous than in the H group. In the descending aorta, the lipid droplets present in the subendothelial connective tissue tended to converge with each other (Figure 7f). The lipid droplets were mainly present in the tunica media’s inner half. However, rare lipid droplets were also noticed in the outer half (Figure 7f).

In the DM group, the thickening of the ascending aorta tunica intima (Figure 7g) was observed due to the proliferation of collagen at the level of the subendothelial connective tissue. The collagen fibers that proliferate in the intimate structure are thin. Rare lipid droplets are observed at this level. The collagen fibers were better represented in the tunica media. The translucent aspect of the elastic blades from the tunica media of this group is much more blurred than in the other groups (Figure 7g,h).

In the structure of the tunica intima of the descending aorta (Figure 7h), large amounts of lipid droplets were not observed, as were in the case of rats from the D group. However, there was observed a localized thickening of the tunica intima. In the structure of the tunica media, the translucent appearance of the elastic blades was visible, and no large amounts of collagen were present.

## 3. Discussion

### 3.1. Weight, Blood Pressure, Glucose, and Lipid Profile

In our study, rats with STZ-induced DM experienced constant weight loss (Figure 1). They presented decreased SBP and DBP (Figure 2) in the first two weeks, followed by elevation of SBP and DBP (Figure 2) in the fourth week. The same group presented the highest levels of glucose (Figure 3) and elevated levels of total cholesterol, LDL-chol, and tryglicerides (Figure 4). The rats with hypertension (H group) presented elevated SBP and DBP (Figure 2) even from the first week. It was observed that D group rats presented increased total cholesterol levels due to increased LDL-cholesterol levels (Figure 4).

Weight loss in rats from the DM group can be explained by the insulin deficit, as streptozotocin leads to the destruction of pancreatic β cells and impaired insulin production [15]. Insulin depletion is correlated with weight loss due to accelerated protein catabolism and diminished protein synthesis [16]. Hyperglycaemia associated with osmotic diuresis was observed to play an important role in the pathogenesis of hypotension and could be the possible explanation for the reduced SBP and DBP observed in the first two weeks in type-1 diabetic rats [17]. The elevated SBP and DBP in the fourth week in the DM group could be explained by sodium retention and a possible abnormal vasodilatory response [18]. The insulin deficit is responsible for the alteration of the lipid profile parameters, as insulin has antilipolytic effects, playing an important role in regulating lipid metabolism. [19]

High SBP and DBP in the H group are due to the fact that L-NAME is an antagonist of nitric oxide synthase (NOS) that slowly releases NO from its guanidino nitro group with a secondary reduction in NO production [20]. The mechanism of L-NAME-induced hypertension is a complex one, and it involves more than a simple decrease in vasorelaxant activity due to inhibition of NO production. Increased activity of the renin–angiotensin–aldosterone system (RAAS) and sympathetic nervous system (SNS) associated with increased prostaglandin secretion and ROS production were described as additional serious factors contributing to the development of L-NAME-induced hypertension [21].

The high cholesterol content diet led to a high serum total cholesterol and LDL-chol in the D group, as the intake of dietary cholesterol is usually associated with an increased intake of saturated fatty acids, which is reported to increase LDL-chol [22]. The increased total and LDL-chol and the lack of change in HDL-chol and triglyceride levels in this group suggests that the phenotype is hyperlipidemia.

### 3.2. Oxidative Stress Parameters

Our results demonstrate that MDA (Figure 5a) was slightly increased by hypertension and significantly elevated by dyslipidemia and type 1 diabetes mellitus. The serum values of NOx (Figure 5b) were reduced in the H and D groups, while rats with DM were associated with the highest levels of NOx. TOS levels (Figure 5c) were increased in all experimental groups, with statistical significance found in the H and DM groups.

Total antioxidant capacity (Figure 5d) and total thiol (Figure 5e) levels were significantly reduced by dyslipidemia and type 1 diabetes mellitus and slightly reduced by hypertension. OSI was significantly increased by hypertension and type 1 diabetes mellitus, and slightly increased by dyslipidemia (Figure 5f).

Malondialdehyde is a degradation product of lipid peroxidation and is widely used as an important indicator of tissue oxidative stress [23]. Elevated MDA in the H group could be explained by the increased tissue lipid peroxidation levels and decreased antioxidant capacity, reflecting the vascular and systemic oxidative stress induced by the L-NAME administration [24]. Hyperlipidemia was observed to cause oxidative stress as LDL-C levels seemed to have a strong positive correlation with MDA levels [25]. Similar results were observed in the D group. Even more, increased LDL-C and MDA serum levels have a positive correlation with vascular inflammation. LDL-C accumulates in arterial walls, inducing differentiation of monocytes into macrophages and the secretion of inflammatory mediators. The inflammation triggers oxidative stress by activating lipoxygenase, myeloperoxidase, and ROS production [25]. In diabetes mellitus, hyperglycemia can increase oxidative stress with increased free-radical formation, resulting in increased lipid peroxidation and MDA production [26], as also observed in our study in the DM group.

Evaluation of the NO production, another biomarker of nitro-oxidative stress, was performed by measuring the serum levels of stable end metabolites such as inorganic nitrites and nitrates (NOx) [27]. NO is the main vasodilatory molecule and is widely used as a biomarker for endothelial dysfunction [28]. Reduced serum NOx levels in the H group are due to the fact that L-NAME is an antagonist of NOS and reduces NO production, thus affecting endothelial and smooth muscle function [20]. It was previously reported that hypercholesterolaemic rats present an endothelial dysfunction in the aorta, with a decreased Ach-induced endothelium-dependent vasorelaxation, and a reduced aortic content of the NO metabolites nitrite and nitrate. Therefore, hypercholesterolemia can induce a reduction of NO levels due to impaired endothelial function [29], as can be seen in the D group. In DM, the nuclear factor κB (NF-κB) pathway is activated by oxidative stress and hyperglycemia. It increases NO production through the expression of the inducible isoform of the nitric oxide synthase (iNOS) gene [30]. This mechanism can explain elevated NOx serum levels in our experimental DM group.

The serum levels of TOS also indicate the degree of oxidative stress. In the H group, TOS is increased because of the elevated oxidation process induced by L-NAME administration [31]. The increased plasma levels of TOS were already observed in rats treated with a high-fat diet [32]. In diabetic animals, hyperglycemia and oxidative-stress induction are responsible for elevated TOS levels [33].

Lower serum levels of TAC are associated with higher serum total peroxide levels, and eventually higher OSI can be explained by excessive oxidative stress via augmentation of lipid peroxidation and protein oxidation produced by L-NAME. The decrease in plasma TAC in rats with dyslipidemia was already demonstrated. It was suggested that hypercholesterolemia-induced oxidative stress causes several changes in erythrocytes, including enhancement of proteins and lipids oxidation and loss of antioxidant power [34]. TAC represents an indicator of the protective effects of antioxidant agents, and it can be used as a new biomarker for the diagnosis and prognosis of DM [35]. The reduction of TAC in the DM group could be attributed to hyperglycemia and lipid peroxidation [35]. The levels of OSI were observed to be elevated in patients with DM [36]. The elevation of OSI in the D and DM groups is due to the imbalance between ROS production and the antioxidant defense that leads to increased TOS and a reduction in TAC [32].

Thiols are mercury-binding mercaptans able to mediate redox-signaling processes to respond to oxidative stress, playing a significant role in mitigating the lipid peroxidative effects of ROS [37]. L-NAME-induced hypertension is associated with low thiol levels [38]. We obtained similar results in the H group. Thiol groups may protect against oxidation of NO molecules by scavenging free radicals and forming nitrosothiols, assuring a prolongation of the NO half-life and potentiating its vasorelaxant effect [38]. It was reported that oxidative stress and elevated levels of oxidized LDL-C are significant factors related to atherosclerosis [39,40]. Hyperglycemia associated with chronic inflammation activates the NF-κB pathway, leading to an increase in oxidative stress [37], which could explain the over-oxidation of thiols in the DM group.

### 3.3. Pro-Inflammatory Cytokines

The tissue levels of TNF-α (Figure 6a), IL-1β (Figure 6b), and IL-6 (Figure 6c) were significantly elevated in the D and DM groups, with the highest levels in the DM group. These pro-inflammatory cytokines were slightly elevated in the H group.

Increased production of these pro-inflammatory cytokines was observed to promote atherosclerosis [41].

TNF-α activates the transcription of NF-kB, regulating the expression of genes involved in inflammation, oxidative stress, and endothelial dysfunction [42]. Induction of ROS production, reduction of NO’s bioavailability, and increased endothelial permeability to circulating blood components and cells are some of the mechanisms that contribute to the pro-atherogenic effects of TNF-α on the endothelium [43]. Apart from the fact that TNF-α contributes to the endothelial barrier disruption, it also regulates vascular permeability to control inflammation. The increased permeability of microvessels allows blood macromolecules and inflammatory cells to enter the injured tissues. This increased permeability of microvessels eventually leads to the subendothelial accumulation of blood lipids and inflammatory molecules and finally to the formation of atherosclerotic plaques [43]. Moreover, new data has shown that TNF-α increases the transcytosis of LDL-chol across human endothelial cells and contributes to early atherosclerosis by enhancing subendothelial retention of LDL-chol in the vascular walls [44].

IL-1β is involved in the initiation of atherosclerosis as it induces an inflammatory response in endothelial cells, which is reflected by the increased expressions of chemokines and adhesion factors. Eventually, IL-1β promotes the accumulation of inflammatory cells in blood vessels and their invasion into the local intima of blood vessels [45]. IL-1β promotes the enhanced expression of IL-1, which stimulates the secretion of other inflammatory mediators, the activation of monocytes and macrophages, and the proliferation and differentiation of vascular smooth muscle cells [46]. Notably, IL-1β plays a vital role in the growth of atherosclerotic plaque [47]. It was previously observed that the selective neutralization of IL-1β elicits a higher plasma level of IL-10 and promotes monocytes to switch to a less inflammatory state in the plasma, favouring the reduction of the size of the plaques without influencing the compensatory outward remodeling in the artery [48].

IL-6 may have pro-atherogenic and anti-atherogenic effects on processes associated with the development and progression of atherosclerosis. Endothelial cell activation, stimulation of vascular smooth muscle proliferation, and platelet activation are the pro-atherogenic effects of this cytokine. The anti-atherogenic effects involve lowering the plasma LDL-chol via upregulated LDL-chol receptor gene expressions [49]. IL-6 mediates the acute phase response, increasing the reactants involved in the formation of atherosclerotic thrombosis, such as C-reactive protein (CRP), fibrinogen, and plasminogen activator inhibitors [47]. It also promotes clot formation and vessel occlusion in atherothrombosis by inducing aggregation and activation of platelets [49]. IL-6 is also a valuable biomarker in predicting future cardiovascular events [42].

### 3.4. Histopathologic Examination

The histopathological changes in the structure of the arterial walls in the H (Figure 7c,d) and D (Figure 7e,f) groups are reversible but are more advanced in the D group than in the H group. In the DM group (Figure 7g,h), the histopathological changes are irreversible, and the proliferation of collagen leads to a reduction of the specific elasticity of the elastic arteries, so that the hemodynamic parameters of the blood in these segments are certainly altered [50]. The particularity of the DM group is that rats from this group developed irreversible pro-atherogenic changes in a short period of time (28 days).

### 3.5. Potential Limitations and Future Research

To our best knowledge, this is the first study that compared the changes in serum oxidative stress biomarkers caused by three cardiovascular risk factors induced experimentally, namely, the following: hypertension, dyslipidemia, and type 1 diabetes mellitus. No other study evaluated and compared the aortic levels of pro-inflammatory cytokines with the above-mentioned cardiovascular risk factors.

L-NAME was administered in the drinking water in the H group, so we could not precisely determine the dose administered to each rat. In the present study, the tissue levels of the pro-inflammatory cytokines were slightly elevated, so maybe a period of time longer than 28 days should be considered whenever the evaluation of aortic pro-inflammatory cytokines in L-NAME-induced hypertension is intended. To create similar standard environmental conditions, rats from the D group had *ad libitum* access to food, so we could not control each rat’s exact amount of cholesterol. Increased glucose levels observed in all study groups might be secondary to mild anesthesia, as the mixture of ketamine and xylazine can mediate the glucoregulatory hormones through stimulation of α2-adrenoceptors [51]. The experimental induction of hypertension, dyslipidaemia, and type 1 diabetes mellitus could not mirror the exact pathogenesis path as in humans since their pathophysiology is often complex and multifactorial. The experimental models of hypertension, dyslipidemia, and type 1 diabetes mellitus can be used in future studies to evaluate different compounds’ antioxidant and anti-inflammatory effects. Future studies should evaluate aortic levels of oxidative stress markers, more pro-inflammatory cytokines, search for other parameters such as microRNAs, and even include immunohistochemistry staining to better understand the implications of these cardiovascular risk factors in atherosclerosis.

## 4. Materials and Methods

### 4.1. Experimental Model

Twenty-eight Wistar-Bratislava white male rats, weighing between 400 and 500 g from the Animal Department of Faculty of Medicine, Iuliu Haţieganu University of Medicine and Pharmacy, Cluj-Napoca, were used in the experiment. Rats were kept in polypropylene cages, acclimatized under standard environmental conditions at 25 ± 2 °C, humidity 50 ± 15%, natural light-dark cycle, and constant room temperature (22 ± 5 °C) at the Pathophysiology Department. Animals had free access to water and food 24 h per day.

The rats were randomly divided into four groups of seven rats/group as follows:

- C = Control group
- H = Hypertension group
- D = Dyslipidemia group
- DM = Diabetes mellitus group

Rats from the C group, the control group, received only standard food and tap water, and no intervention during the whole experiment.

For the induction of hypertension, rats from the H group received L-NAME, (40 mg/kg b.w., body weight dissolved in the drinking water) for 28 days, starting from day one [52].

The rats from the D group received only a hyperlipidemic diet from day one by administering a diet with a high cholesterol content (2%).

Type 1 diabetes mellitus was induced on day one by intraperitoneal administration of a single dose of streptozocin (65 mg/kg b.w.) freshly prepared 0.01 M citrate buffer (pH = 4.5) [53]. Forty-eight hours later, blood samples were taken from the retro-orbital plexus, and glucose levels were measured with a glucometer (VivaChek Biotech (Hangzhou, China)). Animals with blood serum glucose levels higher than or equal to 200 mg/dL were considered to have DM [54]. Two units of a mixture of insulin (a rapid-acting and an intermediate-acting effect) were administered daily at 2 PM to prevent acute complications of type 1 DM and death.

Animals from all groups had free access to food and water *ad libitum*.

### 4.2. Drugs and Chemicals

L-NAME (N omega-nitro-L-arginine-methyl) and STZ (streptozotocin) were purchased from Sigma-Aldrich (St. Louis, MI, USA).

Dyslipidaemia was induced with a diet prepared from natural ingredients based on 90 g standard diet, to which were added 2 g of cholesterol and 8 g of saturated fat. The following values were recorded by determining the main nutritional parameters of the diet: protein 22.84 g, cellulose 4.38 g, and lipids 11.65 g in 100 g diet with a content of 91.35 g dry substance. The diet was purchased from Cantacuzino Institute (Bucharest), Declaration of Conformity no. 31 from 23 February 2021.

NovoMix 70 FlexPen 100 U/mL (an insulin analogue) with both a rapid-acting and an intermediate-acting effect, in a 70/30 ratio, was purchased from a local pharmacy.

### 4.3. Blood Pressure Measurement

Systolic blood pressure (SBP) and diastolic blood pressure (DBP) were measured in non-anesthetized rats from all four groups using the tail-cuff plethysmography of a Biopac MP36 device with the NIBP200A extension on days 0 (week 0), 7 (week 1), 14 (week 2), 21 (week 3), and 28 (week 4), as previously described [55]. Three measurements were performed for each rat, and the mean value was reported.

### 4.4. Blood Samples and Serum Analysis

On day 28, after fasting 12 h, under mild anesthesia with ketamine and xylazine, blood samples were collected from the retro-orbital plexus of each rat. After that, the rats were sacrificed with an overdose of anesthetics.

Serum levels of the following oxidative stress biomarkers: malondialdehyde (MDA), the indirect assessment of NO synthesis (NOx), total oxidative status (TOS), total antioxidative capacity (TAC), and total thiols (SH) were assessed with a Jasco V-530 UV–Vis spectrophotometer (JASCO International Co. Ltd., Tokyo, Japan) [56].

The serum levels of total cholesterol, LDL-chol, high-density lipoprotein cholesterol (HDL-chol), and triglycerides were determined using commercially available kits, through a spectrophotometric method with an automatic analyser by Applied Biosystems (BioSystem Costa Brava, Barcelona, Spain).

### 4.5. Histopathological Examination

Right after blood samples were taken, rats were sacrificed, and 4 mm thick slices were collected from the base of the heart—from the ascending aorta and the descending aorta—the thoracic segment. The samples collected were fixed in a Stieve mixture for 24 h, dehydrated in ethyl alcohol, clarified with butyl alcohol, and embedded in paraffin. Sections with a thickness of 5 µm were made of paraffin blocks and colored by Goldner’s trichrome method. Histological preparations were examined under an optical microscope (Olympus BX41), and photographs were taken (Olympus SC180 camera and Olympus cellSens Entry software for image acquisition).

### 4.6. Tissue Homogenate and Pro-Inflammatory Cytokines

Fragments from the ascending aorta were taken, weighed, and homogenized in four volumes of phosphate-buffered saline solution. The homogenization was made using an automated Witeg Homogenizer (HG-15D, Wertheim, Germany) at 27,000 rpm. Right after the homogenate was centrifuged (15,000 rpm at 4 °C for 15 min), the clear supernatant was used for pro-inflammatory cytokine analysis. The tissue levels of TNF-α, IL-6, and IL-1β, were assessed using the ELISA technique (Stat Fax 303 Plus Microstrip Reader, Minneapolis, USA), with commercially available kits (rat TNF-α, IL-6, and IL-1β ABTS ELISA development kits, PeproTech EC, Ltd., London, UK).

### 4.7. Statistical Analysis

Statistical analysis was done with IBM SPSS Statistics (Trial version, IBM, New York, Armonk, USA) to evaluate the effects of hypertension, dyslipidemia, and type 1 diabetes mellitus on the biomarkers being assessed. Data were presented as mean and standard deviation. Kruskal–Wallis test followed whenever appropriate by post-hoc analysis was used to evaluate differences between groups. Weight and blood pressure were compared for the same group with Friedman test, and data were presented using line charts with circles representing the mean value of each group, and the bottom and upper lines representing one standard deviation. In figures illustrating glucose, lipid profile, oxidative stress parameters, and pro-inflammatory cytokines data were represented as individual values (circles) and median (as a line) [57]. The statistical tests were two-sided at an estimated significance level of 0.05, and a *p*-value < 0.05 was considered statistically significant.

## 5. Conclusions

Hypertension and dyslipidemia can induce reversible pro-atherogenic changes consisting of an accumulation of lipid droplets in the subendothelial connective in a short period of time. Type 1 diabetes mellitus is the cardiovascular risk factor that primarily alters the oxidative stress parameters, elevates the pro-inflammatory cytokines’ aortic levels, and induces irreversible pro-atherogenic changes in the aortic wall. Experimental models of hypertension, dyslipidemia, and type 1 diabetes mellitus can be used to evaluate the antioxidant and anti-inflammatory effects of different compounds in the atherosclerotic process.

## Data Availability

The data analysed in the experiment can be obtained upon reasonable request addressed to Paul-Mihai Boarescu (e-mail: boarescu.paul@umfcluj.ro).
